# TGF-β Enhances Phosphate-Driven Calcification of Human OA Articular Chondrocytes

**DOI:** 10.1007/s00223-025-01365-x

**Published:** 2025-04-02

**Authors:** Roderick H. M. J. Stassen, Guus G. H. van den Akker, Marjolein M. J. Caron, Don A. M. Surtel, Andy Cremers, Lodewijk W. van Rhijn, Tim J. M. Welting

**Affiliations:** 1 Laboratory for Experimental Orthopedics, Department of Orthopedic Surgery, Maastricht University, Maastricht, The Netherlands; 2 Laboratory for Experimental Orthopedics, Department of Orthopedic Surgery, Maastricht University Medical Center +, Maastricht, The Netherlands

**Keywords:** Chondrocyte, Osteoarthritis, Calcification, Transforming-growth factor β, Phosphate

## Abstract

The pathological relevance of articular cartilage calcification in osteoarthritis (OA) is becoming increasingly evident. We are only beginning to understand the pathobiological mechanisms that contribute to articular cartilage calcification in OA. How molecular environmental factors interact with calcification mechanisms is poorly explored. In this study, we developed an in vitro phosphate-driven calcification model for human OA articular chondrocytes, in which these cells are cultured in the presence of calcification medium containing adenosine triphosphate (ATP) and β-glycerophosphate (BGP). We employed this model to investigate the role of transforming growth factor β (TGF-β) in chondrocyte calcification. Chondrocyte culture in calcification medium resulted in mineral nodule formation over a time course of 7 days. The presence of calcium and phosphate deposition in these nodules was validated with von Kossa staining, scanning electron microscopy–energy-dispersive X-ray spectroscopy (SEM–EDX), and colorimetric calcium and phosphate assays. Supplementation of calcification medium with TGF-β resulted in enhanced nodule formation with a different morphology and changed the expression of extracellular matrix-related genes such as collagen type I and III. In conclusion, we developed a new in vitro model for human OA articular chondrocyte calcification, in which we demonstrated a pro-calcifying role for TGF-β. This in vitro model may be used as a basis to aid the investigation of the influence of environmental factors on chondrocyte calcification and the development of new anti-calcification disease-modifying osteoarthritis drugs.

**Supplementary Information:**

The online version contains supplementary material available at 10.1007/s00223-025-01365-x.

## Introduction

Osteoarthritis (OA) is a degenerative joint disease that affects all tissues of the synovial joint. OA has an increasing prevalence and no curative treatment is currently available. Aside from synovial inflammation and articular cartilage degeneration, calcification of the synovium, meniscus, and articular cartilage are observed in OA [1]. Calcium-containing crystals are found both in the superficial and in the deeper zones of OA articular cartilage, and pathological calcification occurs in a top-down as well as a bottom-up manner [2].

Over the last decades, several mechanisms have been proposed to explain pathological calcification of OA articular cartilage, including membrane vesicles (MV) and apoptotic bodies. These have been shown to function as early mineralization nuclei, which allow the formation of amorphous calcium phosphate precursors [1]. Release of these precursors into the cartilage extracellular matrix (ECM) subsequently facilitates further crystal growth. In addition, it is hypothesized that an imbalance in both calcium and phosphate ions contributes to calcification [3]. Phosphate homeostasis is maintained by balancing the inorganic pyrophosphate (PPi) and inorganic phosphate ratio (Pi). PPi is considered as an important inhibitor of calcification [4], while Pi is necessary for the deposition of hydroxyapatite [5]. An imbalance favoring supraphysiological concentrations of Pi results in the formation of Posner clusters, which in turn lead to the formation of amorphous calcium phosphate particles [6].

The human body utilizes a large variety of transporters and enzymes in order to tightly control the phosphate balance in extracellular fluids. Enzymatic hydrolysis resulting in PPi formation is controlled by soluble and membrane bound pyrophosphatases. A membrane-bound pyrophosphatase expressed in articular chondrocytes and linked to calcification is ectonucleotide pyrophosphatase/phosphodiesterase 1 (ENPP1) [7]. This type II transmembrane glycoprotein has been shown to degrade a large variety of substrates, including adenosine triphosphate (ATP) and adenosine diphosphate (ADP), into PPi [8]. Furthermore, intra- and extracellular levels of PPi are regulated by ATP transporters Ankh and Abcc6 [9]. Their role in calcification has become clear in relation to calcification pathologies. Ankh mutations result in the development of chondrocalcinosis [10], while mutations in the Abcc6 result in development of pseudoxanthoma elasticum [11]. In contrast, Pi generation by chondrocytes is partially regulated by alkaline phosphatase (ALP). Pi is generated from various sources such as ATP, ADP, and PPi [12]. Intracellular transport of Pi is regulated by the SLC20 family. The SLC20 family are type III sodium-coupled Pi cotransporters; this results in symport of sodium and Pi in a ratio of 2:1 [13]. These systems together facilitate tight control of the PPi/Pi balance.

In addition to the PPi/Pi balance, the cartilage ECM composition plays an important role in the development of pathological cartilage calcification. Both collagens and proteoglycans have been shown to possess calcification-regulating capacities, especially collagen type I and X [14, 15]. In contrast to collagens, the larger proteoglycans found in articular cartilage are considered as mineralization inhibitors, due to sterical hindrance and charge distribution [16].

As a result of the significant role of the ECM in the calcification propensity of articular cartilage, it can be postulated that microenvironmental factors play a role in regulating the mineralization of the cartilage ECM. One of the best described morphogens with respect to cartilage matrix synthesis is TGF-β, which has an ambivalent function in articular cartilage homeostasis [17]. In normal cartilage physiology, TGF-β was shown to be essential for maintenance of a healthy cartilage homeostasis, among others by inhibition of chondrocyte hypertrophy and MMP-13 expression [18]. However, in OA, TGF-β has a pathological role, resulting from predominant ALK-1 signaling [17] and loss of the protective effect of SMAD2/3 activity [19]. Besides the change in signaling route, the timing and the dosage of TGF-β appear to be of key importance. For example, stimulation of chondrocytes with TGF-β resulted in increased expression of collagen type II and aggrecan [20], while longer stimulations resulted in increased expression of collagen type I and X [21].

Previously, the effect of TGF-β on calcification was studied in tissues with intrinsic physiological calcification properties, such as bone and teeth. Studies have dissected the role of TGF-β in every part of the bone formation cascade, where in the later stages of osteoblast differentiation, TGF-β blocks mineralization [22]. In contrast, a pro-mineralizing effect of TGF-β has been observed in ameloblasts. Conditional knock-out of TGF-βRII in mice showed decreased mineralization of enamel and thinner crystallites [23], indicating a pro-mineralizing role for TGF-β.

Previously, we showed that TGF-β signaling plays an important role in basic calcium-phosphate (BCP) crystal-driven secretion of pro-inflammatory IL-6 by articular chondrocytes [24]. Nevertheless, the role of TGF-β in chondrocyte mineralization remains to be uncovered. In this study, we developed a phosphate-driven in vitro mineralization model for human OA articular chondrocytes (HACs) in order to evaluate the effect of TGF-β on the mineralization properties of OA chondrocytes.

## Materials and Methods

### Isolation and Culture of Primary Human OA Articular Chondrocytes

Human OA articular chondrocytes were isolated from surgical waste material (Medical Ethical permit METC2022-3235) as previously described [24]. In short, cartilage was dissected from the bone and digested in collagenase type II solution (300 U/ml) at 37 ⁰C. After overnight digestion, the cell suspension was strained using a 70-μm filter and cultured in DMEM/F12 (Gibco) containing 10% fetal calf serum (FCS), 1% antibiotic/antimycotic (Gibco), and 1% non-essential amino acids (NEAA; Gibco) until passage 1 to minimize dedifferentiation. HACs were cultured in humidified atmosphere at 37 ⁰C and 5% CO_2_.

### Induction of Calcification

HACs derived from 6 donors (average age 62 ± 18.4 years; BMI 31.9 ± 3.6 kg/m^2^) were pooled, seeded at 30.000 cells/cm^2^ , and allowed to attach overnight. Subsequently, cells were cultured in proliferation medium supplemented with 1 mM ATP and 10 mM BGP (calcification medium). To investigate the role of TGF-β, calcification medium was supplemented with 10 ng/ml rhTGF-β3 (R&D) in the presence or absence of 5 μM ALK5 kinase inhibitor SB5050124 (Selleck Chemicals). DMSO was used as the appropriate vehicle control. Media were refreshed every 2 days and cells were cultured up to 7 days.

### Calcium and Phosphate Determination

Cells were washed twice with 0.9% NaCl and overlaid with 100 µl 0.1 M HCl solution (VWR Chemicals). Crystal hydrolysis was allowed for 3 h and confirmed by light microscopy. Subsequently, these hydrolysates were used to determine calcium and phosphate content. Calcium content was determined with a colorimetric calcium assay (Randox) according to manufacturer’s protocol. In brief, 5 µl lysate was incubated with 250 µl O-cresolphthalein complexone and absorbance was measured at 560 nm with the use of a Multiskan™ FC microplate spectrophotometer (Thermo Scientific). Phosphate present in the lysate (5 µl) was incubated with a chromogenic complex (30 µl) according to the manufacturer’s protocol (Sigma) and absorbance was determined at 650 nm.

### Von Kossa Staining and Quantification

After 7 days, cells were washed twice with 0.9% NaCl, air-dried, and fixed with 4% formaldehyde (VWR Chemicals) for 1 h on a linear shaker. Next, cells were washed once with MiliQ and incubated with 1% silver nitrate solution (VWR chemicals) for 30 min in the dark. The silver nitrate solution was removed and cells were washed five times with MiliQ. Development of the stain was done for 30 min under UV light in a ChemiDoc (Bio-Rad). Specific staining was confirmed by microscope and 4 high power field images were obtained to determine the calcified area. Quantification was performed using particle analysis with ImageJ (Version 1.54f).

### Scanning Electron Microscopy with Energy-Dispersive X-Ray Spectroscopy

Chondrocytes were seeded in a 24-well plate at a density of 30.000 cells/cm^2^ and cultured under calcifying conditions for 7 days. Subsequently, cells were washed twice with 0.9% NaCl and fixed with 4% formaldehyde solution for 1 h. Next, the bottom of the well plate was removed and placed on a SEM specimen stub. Images and elemental analysis were obtained with a JSM-IT200 InTouchScope™ Scanning Electron Microscope (JEOL).

### Gene Expression Analysis

Cells were lysed in TRIzol™ Reagent (ThermoFisher Scientific). Phase separation was performed and RNA was precipitated with isopropanol at − 20 ⁰C overnight. The sample was centrifuged at 20,000 × *g* for 60 min and the pellet was washed with 80% ethanol. The RNA pellet was resuspended in RNAse-free MiliQ water and concentration was determined with use of a Nanodrop One (Thermo Scientific). cDNA was prepared using the random hexamer primer method using 500 ng of RNA in a Biometra TRIO (Analytik jena) according to the following protocol: 6 min at 72 ⁰C followed by 1 h and 5 min at 37 ⁰C and 5 min at 95 ⁰C. RT-qPCR reactions were performed using 12 µl of Takyon NO ROX SYBR MasterMix dTTP blue (Eurogentec) supplemented with 6 ng of cDNA and 0.3 µM of both forward and reverse primers which resulted in a reaction volume of 15 µl. Primers are listed in Supplementary Table 1. Amplification was performed in a Bio-Rad CFX96 Real-Time PCR Detection system with the following protocol: 10 min denaturation at 95 ⁰C, 40 amplification cycles comprising 15 s at 95 ⁰C and 1 min at 60 ⁰C. Primers used in this study are shown in supplementary Table 1. Gene expression analysis was done using the standard curve method and data were normalized to *PPIA*.

### Enzyme-Linked Immunosorbent Assay

The protein concentration of IL-6 was determined in the culture medium using an enzyme-linked immunosorbent assay. Samples were analyzed in compliance with the manufacturer’s protocol (R&D). Absorbance was determined with a Multiskan™ FC microplate spectrophotometer (Thermo Scientific) at 450 nm.

### Statistical Analysis

Chondrocytes from six distinct donors were used in a pooled strategy to minimize interpatient variation and the same pool was used for all experiments. All experiments were performed in at least triplicates. Normal distribution of the data and equal variance were assumed based on the sample size. Statistical significance was determined using a two-tailed unpaired Student’s *T*-test. Observations were considered significant when *P* value ≤ 0.05.

## Results

### Phosphate Imbalance Induces Calcification of ECM Produced by HACs

**Fig. 1:**
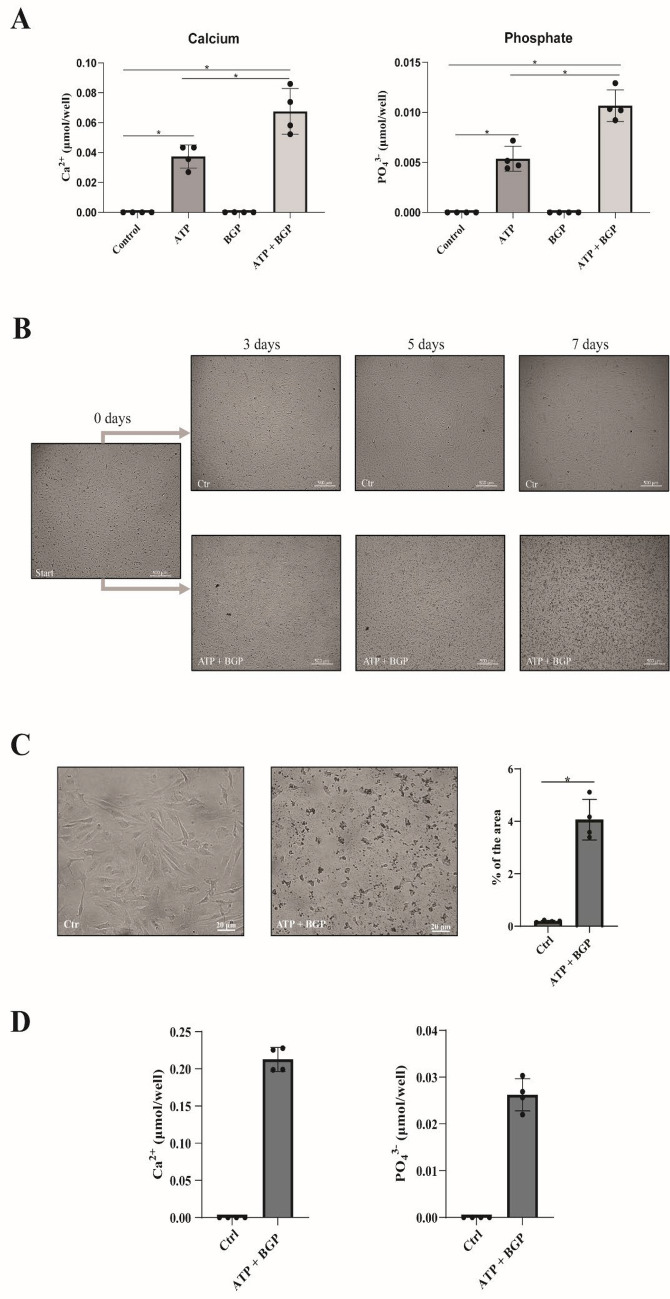
Introduction of phosphate imbalance results in calcification of human OA articular chondrocytes. **a** Pooled human OA articular chondrocytes (*n* = 6) were pooled and cultured in the presence of solely ATP and BGP and in the combination of ATP and BGP. After 7 days, calcium and phosphate were determined in the lysate to determine calcium-containing crystal formation induced by these phosphate donors. **b** A pool of human OA articular chondrocytes (*n* = 6) were cultured in the presence of 1 mM ATP and 10 mM BGP over the time course of 7 days. Microscopic images obtained at 3, 5, and 7 days. **b** Von Kossa-stained human OA articular chondrocytes after culturing in calcification medium for 7 days. Positive staining was quantified with ImageJ (*n* = 4). **c** Colorimetric determination of calcium and phosphate content after crystals hydrolysis after 7 days of culture (*n* = 4). Data in bar graphs are presented as Mean ± SD. Statistical significance was determined with an unpaired Student’s *T*-test. * *P* value < 0.05. Introduction of phosphate imbalance resulted in OA-related crystal species formation and altered gene expression levels of phosphate transporters and generating enzymes

An imbalance in synovial fluid phosphate levels is thought to contribute to cartilage calcification in OA [3]. To develop an in vitro HAC calcification model based on this imbalance, we used two phosphate donors: ATP and BGP. Calcification of HAC cultures in the presence of both ATP and BGP showed increased mineral formation compared to either ATP or BGP-supplemented culture medium (Fig. 1a). As early as 3 days after the first exposure to this medium, small cell-associated mineral deposits could be observed in the culture wells, which increased in number over consecutive days (Fig. 1b). This visual increase in nodule formation appeared to occur in concordance with a more apoptotic cellular morphology which could be confirmed by LDH activity measurements (Supplementary Fig. 1). To determine whether these nodules were indeed calcium-containing crystals, we performed von Kossa stainings (Fig. 1c). A significant increase in the von Kossa-positive area was found. In addition, increased calcium and phosphate contents were detected in crystal hydrolysates from the ATP + BGP condition (Fig. 1d). The formation of these nodules occurred in a cell-dependent manner since no nodules were formed in the cell-free system (Supplementary Fig. 2). Together, this confirmed development of calcium-containing crystals by HACs in the ATP + BGP-supplemented culture medium.

**Fig. 2:**
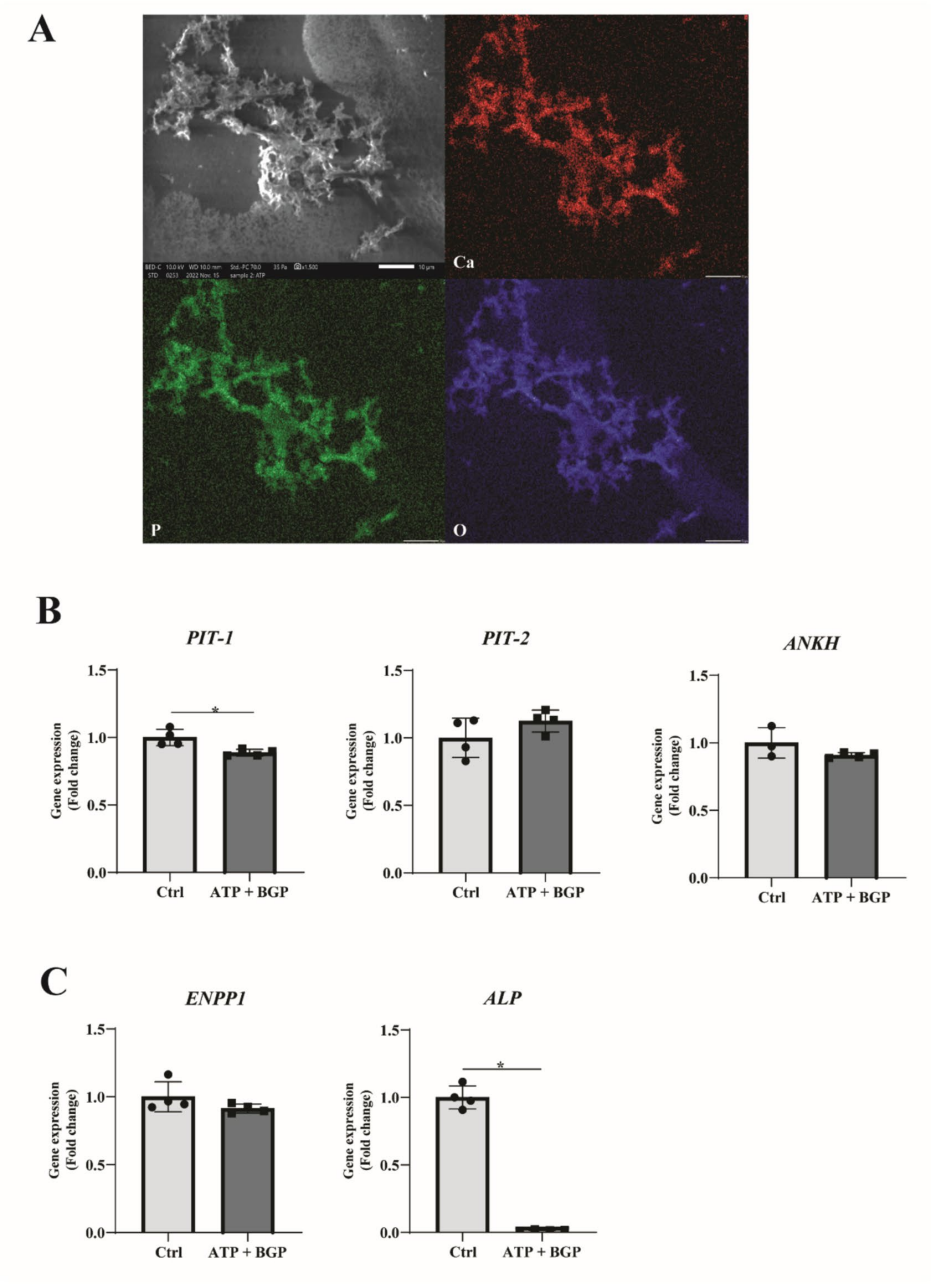
Phosphate-induced calcification of human OA articular chondrocytes alters gene expression levels of phosphate homeostasis transporters and enzymes. **a** SEM–EDX analysis of calcification nodules deposited after 7 days of culture. Identification of calcium, phosphorus, and oxygen atoms indicates the presence of calcium phosphate mineral deposition. **b** Human OA articular chondrocyte gene expression analysis of phosphate transporters Pit-1, Pit-2, and ANKH was evaluated by RT-qPCR after 7 days in calcification medium (*n* = 4). C) Gene expression analysis performed on human OA articular chondrocytes by RT-qPCR for phosphate-generating enzymes ENPP1 and ALP (*n* = 4). Data are presented as Mean ± SD. Statistical significance was determined with an unpaired Student’s *T*-test. * *P* value < 0.05

A great variety of biologically relevant calcium-containing crystals can be found in the human body. The type of crystal formed is highly dependent on environmental factors such as pH and temperature [25]. Elemental analysis was therefore used to determine the crystal species formed in our in vitro model. Samples were prepared for SEM–EDX analysis, after which crystal locations were determined in the sample and area mapping was performed. Thepresence of Ca, P, and O was detected in nodules, which indicates the presence of calcium phosphate-containing crystals (Fig. 2a). Mineral species characterization was performed using spot analysis and showed a Ca:P ratio of 1: 1.41 (Supplementary Fig. 3). This ratio indicates the formation of amorphous calcium phosphate (ratio ranging between 1.2 and 2.2) or α/β-Tricalcium phosphate (ratios of both are 1.5) [26]. Despite the presence of ATP, we did not find any evidence for the presence of calcium pyrophosphate species, typically formed due to increased extracellular ATP levels [27].

To characterize the potential role of phosphate transport and generation in our in vitro model, gene expression levels were examined. After 7 days of chondrocyte calcification, we observed a small (FC = 1.13), yet statistically significant decrease in *SLC20A1* expression, while the expression of *SLC20A2* and *ANKH* remained unaltered (Fig. 2b). Additionally, we determined the gene expression levels of PPi- and Pi-producing enzymes *ENPP1* and *ALPL*. Gene expression levels of *ENPP1* did not change under mineralizing conditions, while *ALPL* gene expression was significantly decreased (Fig. 2c). These results indicate a potential limited role with respect to phosphate transporters, while a particular imbalance was induced between the two main PPi- and Pi-generating enzymes.

### Phosphate-Induced Chondrocyte Calcification Reduces the Collagen Gene Expression and Increases the Expression of Pro-Inflammatory Molecules and MMPs

**Fig. 3:**
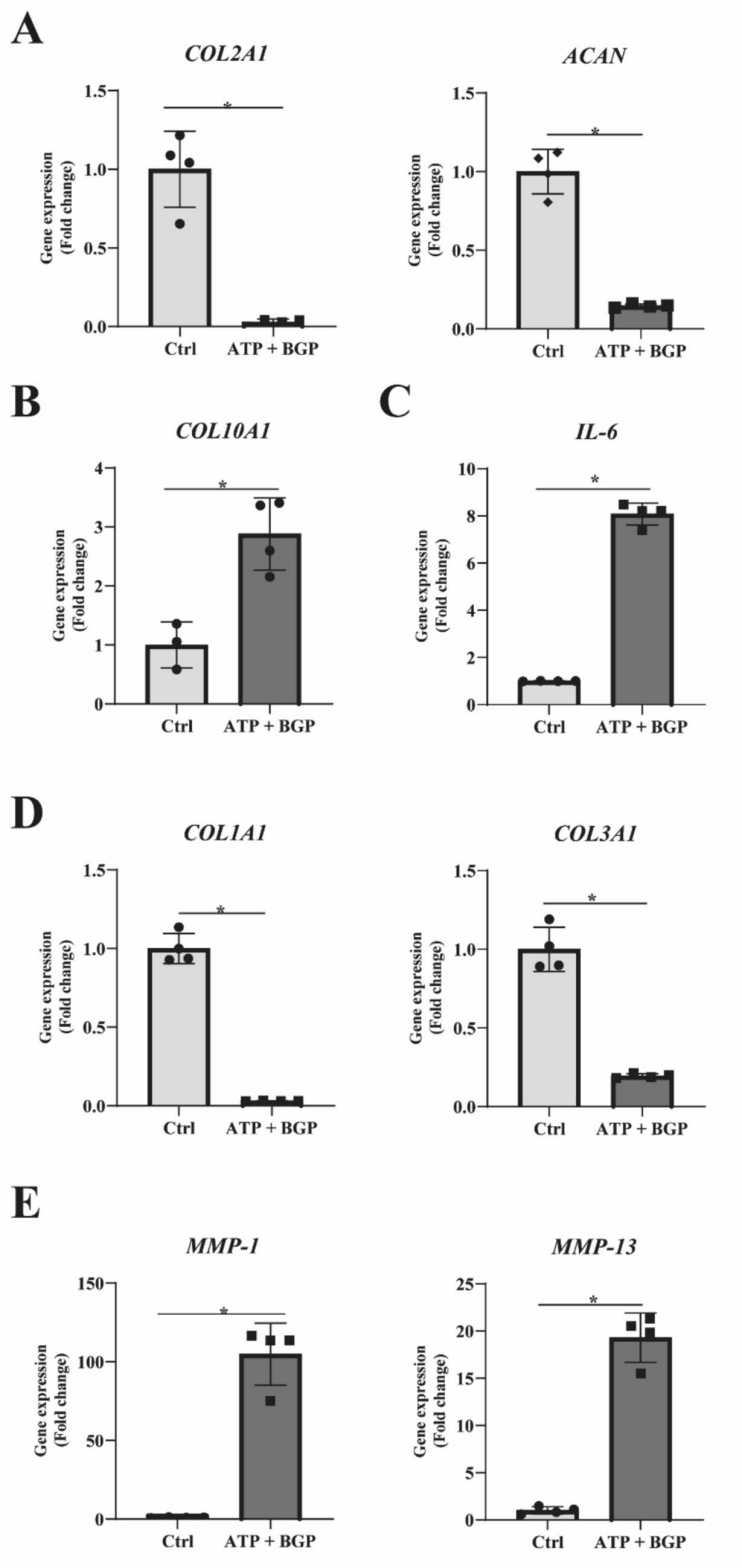
Human OA articular chondrocytes cultured under calcifying conditions decrease collagen gene expression levels accompanied by an increase in pro-inflammatory and matrix degrading. **a** Gene expression analysis of a pool of human OA articular chondrocytes (*n* = 6) with respect to extracellular matrix components COL2A1 and ACAN. **b** Hypertrophic marker COL10A1 expression as a result of calcification. **c** IL-6 gene expression levels determined by RT-qPCR. **d** Fibrocartilage-associated collagen gene expression levels of COL1A1 and COL3A1. **e** Gene expression levels of important matrix degrading enzymes MMP-1 and MMP-13. All gene expression levels (*n* = 4 for each measurement) were determined after the 7-day culture period in calcifying conditions. Data are presented as Mean ± SD. Statistical significance was determined with an unpaired Student’s *T*-test. *P value < 0.05

Apart from the availability of relevant ions to support calcification, the composition of the ECM plays a key role. Several cartilage ECM components, such as collagen type I and type X, have been shown to facilitate calcification by providing nucleation sites [14, 15]. We therefore determined the effect of the calcification environment on pro-inflammatory, chondrogenic, hypertrophic, catabolic, and fibrotic gene expression. The calcification medium significantly decreased the expression of *COL2A1* and *ACAN* (Fig. 3a) and increased the expression of *COL10A1* (Fig. 3b). The phosphate imbalance in the calcification medium resulted in increased gene expression levels of *IL-6* (Fig. 3c). Exposure to calcifying conditions resulted in decreased the expression of *COL1A1* and *COL3A1* (Fig. 3d). In contrast, calcifying conditions induced the expression of *MMP-1* and *MMP-13* (Fig. 3e).

These results demonstrate major OA-related phenotypic changes in HACs under calcifying conditions. This phenotypic response includes an imbalance in ECM homeostasis and an elevated pro-inflammatory state.

### TGF-β Enhances Phosphate-Driven Calcification of Extracellular Matrix Produced by Human Articular Chondrocytes

**Fig. 4:**
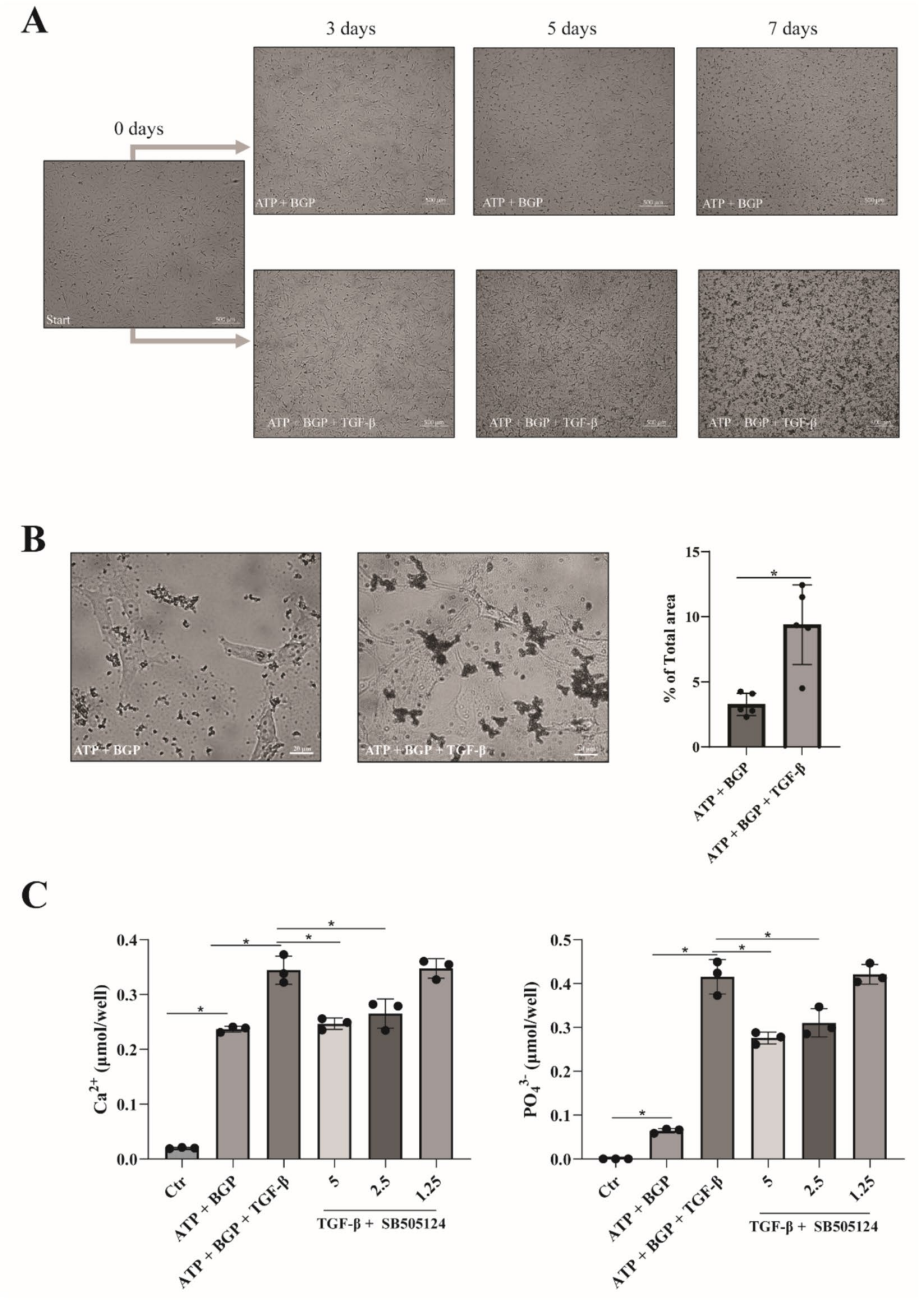
TGF-β-enhanced nodule formation in human OA articular chondrocytes under phosphate-driven calcifying conditions. **a** Human OA articular chondrocytes were cultured in the presence of ATP (1 mM) and BGP (10 mM), with or without supplementation of rhTGF-β. Microscopic images were obtained after 0, 3, 5, and 7 days of culture. Scale bar indicates 500 μm. **b** Von Kossa-stained 7-day cultures in the presence or absence of TGF-β. High-power magnifications were quantified with use of ImageJ. **c** Colorimetric quantification of calcium and phosphate in crystal lysate. Lysate was obtained after 7 days of culture with or without the addition of TGF-β and SB505124 (5, 2.5, and 1.25 μM). Data are presented as Mean ± SD. Statistical significance was determined with an unpaired Student’s *T*-test. * *P* value < 0.05

After establishing a model for phosphate-driven calcification of HACs, we investigated the role of TGF-β in this process. Supplementation of the calcification medium with TGF-β resulted in enhanced mineral nodule formation, which appeared larger in size than in control cultures (Fig. 4a). In TGF-β conditions, nodules became readily visible after 3 days of culture and appeared larger in size. Increased von Kossa positivity (Fig. 4b) in combination with a higher calcium and phosphate content indicated pro-calcifying activity of TGF-β (Fig. 4c). This effect was not mediated by an increased apoptotic rate of the chondrocytes (Supplementary Fig. 1). The increase in TGF-β-mediated calcification could be inhibited by the ALK5 kinase inhibitor SB505124 in a dose-dependent manner (Fig. 4c). Overall, these results support a pro-mineralizing role of TGF-β3 on phosphate-driven calcification of chondrocytes.

### TGF-β Changes Mineral Nodule Structure and Increases SLC20A1 and ENPP1 Expression

**Fig. 5:**
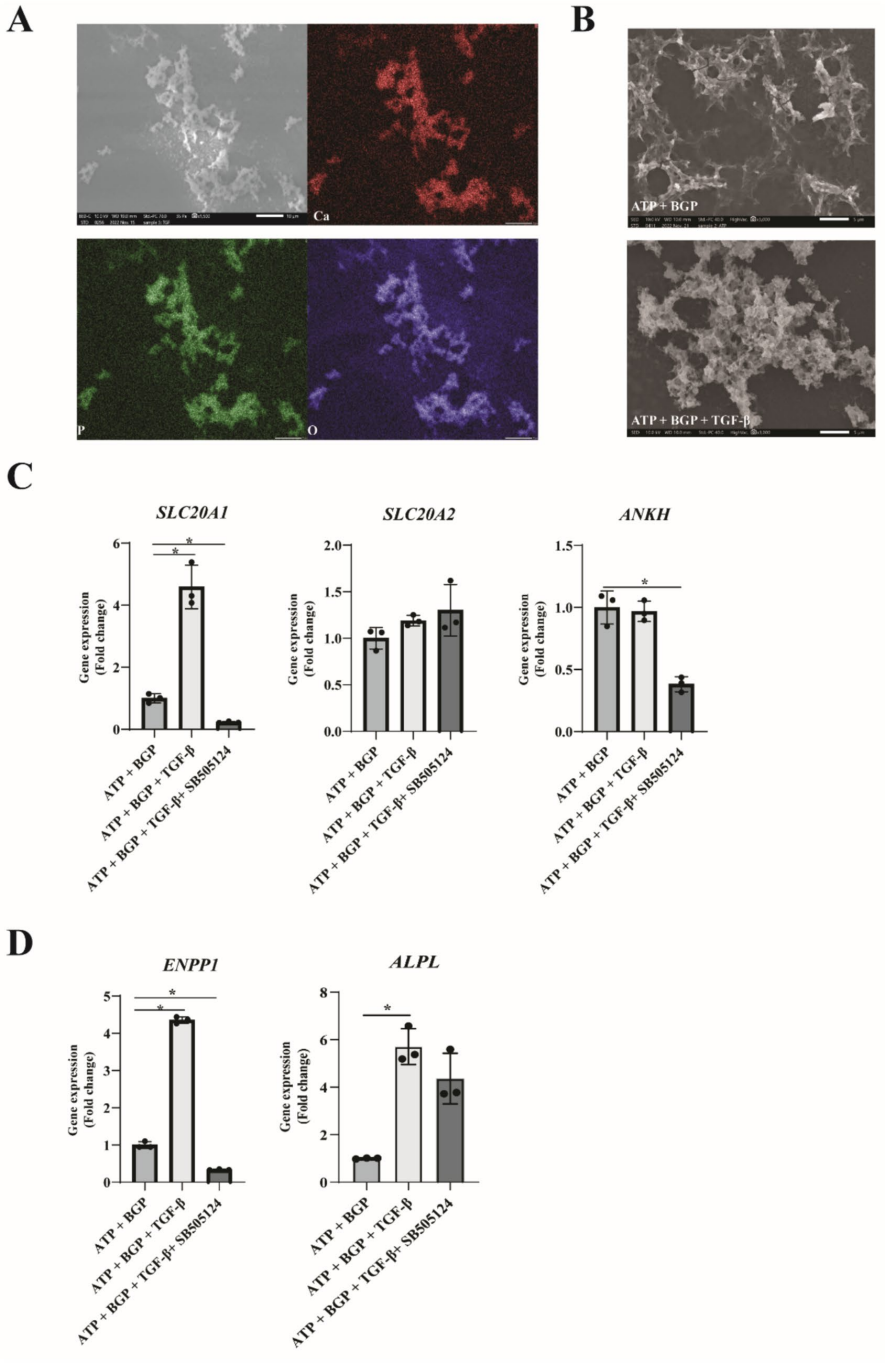
TGF-β increased gene expression levels of phosphate transporters and free phosphate-generating enzymes under phosphate-driven calcifying conditions. **a** SEM–EDX analysis of calcification nodules showing the presence of calcium, phosphorus, and oxygen presence in mineral nodules formed. **b** Scanning electron microscopic image obtained from calcifying conditions in the presence or absence of TGF-β supplementation. Images are obtained at 10 kV and a magnification of 3000 × . **c** Gene expression analysis determined by RT-qPCR investigating expression levels of phosphate transporters *Pit-1,* *Pit-2,* and *ANKH*. **d** Human OA articular chondrocyte gene expression levels of free phosphate-generating enzymes *ENPP1* and *ALP*. Experiments were performed in triplicate. Data are presented as Mean ± SD. Statistical significance was determined with an unpaired Student’s *T*-test. *P value < 0.05

To confirm our TGF-β-related light microscopy and von Kossa observations, we examined these crystals in more detail. First, we determined the presence of Ca, P, and O atoms in the mineral nodules. SEM–EDX demonstrated that these elements are present in the nodules that were generated under TGF-β conditions (Fig. 5a). SEM images obtained at a magnification of 3000 × uncovered a clear difference in mineral nodule morphology between the conditions. Minerals formed in the control calcifying conditions show a well-aligned and clearly defined crystal structure (Fig. 5b). Centrally in the nodules, a well-defined circular structure is observed, while on the edges, the structures are stalk-like. TGF-β-dependent mineral nodules were observed as cauliflower-like micro-organized structures. Spot element analysis further supported a clear difference between the minerals formed in the two conditions. The Ca:P ratio of nodules formed in the control calcifying condition was 1:1.41, while in the TGF-β-supplemented condition, this ratio was 1:1.05 (Supplementary Fig. 4). Based on the Ca:P ratio, the control calcification medium supported the formation of amorphous calcium phosphate (ratio ranging between 1.2 and 2.2) or α/β-tricalcium phosphate (ratios of both are 1.5), while supplementation with TGF-β resulted in the formation of either brushite or monetite (both having a Ca:P ratio of 1) [26].

To better understand the differential chondrocyte response associated with the TGF-β-induced mineralization, we measured gene expression of phosphate transporters and phosphate-generating enzymes. Cultures in calcification medium supplemented with TGF-β presented with increased expression of *SLC20A1*. No TGF-β-driven change was observed for *SLC20A2* or *ANKH* (Fig. 5c). PPi and Pi-generating enzymes *ENPP1* and *ALPL* were both significantly increased in the presence of TGF-β (Fig. 5d). The TGF-β-mediated increase of *SLC20A1* and *ENPP1* could be inhibited by SB505124, supporting a role for TGF-β signaling in the TGF-β-mediated expression of both genes.

### TGF-β Supplementation Alters Gene Expression of Extracellular Matrix Maintenance Genes

**Fig. 6:**
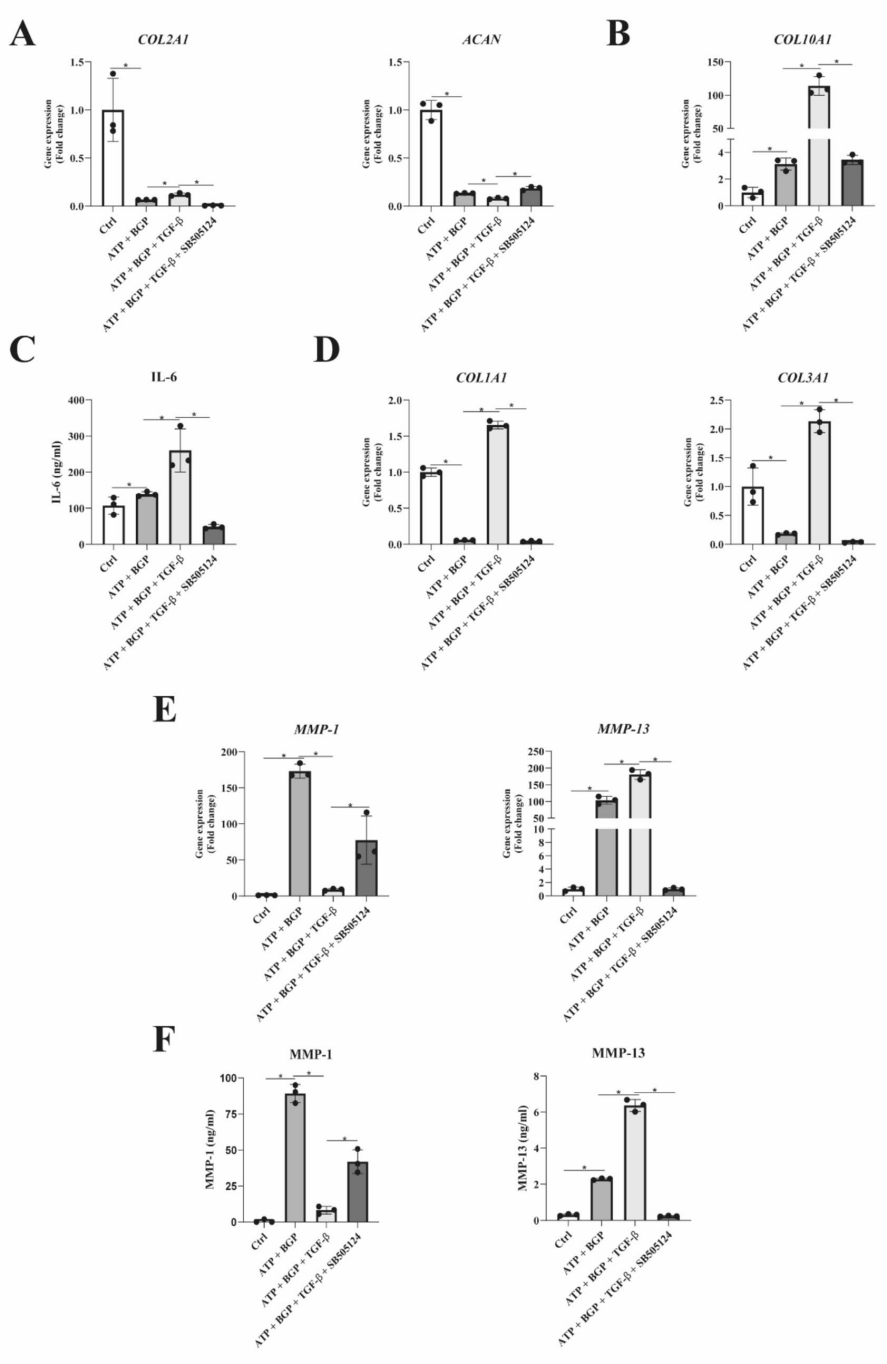
The presence of TGF-β under phosphate-driven calcifying conditions enhances gene expression levels of calcification-related collagens and downregulates matrix degrading proteins. **a** Gene expression analysis of a pool of human OA articular chondrocytes (*n* = 6) with respect to extracellular matrix components *COL2A1* and *ACAN* normalized to calcifying conditions. **b** Hypertrophic marker *COL10A1* expression as a result of calcification in the presence and absence of TGF-β and SB505124. **c** Secreted protein levels of IL-6 determined in culture media. **d** Fibrocartilage-associated collagen gene expression levels of *COL1A1* and *COL3A1*. **e** Gene expression levels of important matrix degrading enzymes *MMP-1* and *MMP-13*. **f** Secreted levels of total MMP-1 and MMP-13 determined in culture media under calcifying conditions in the presence or absence of TGF-β and SB505124. All gene expression levels and secreted protein (*n* = 3) were determined after the 7-day culture period with respect to the different conditions. Data are presented as Mean ± SD. Statistical significance was determined with an unpaired Student’s *T*-test. *P value < 0.05

The composition of the ECM plays an important role in its calcification potential [28]. Therefore, we determined the effect of TGF-β on the expression of genes involved in chondrocyte ECM maintenance. Compared to the control calcification condition, the addition of TGF-β resulted in increased expression of *COL2A1*, but a decreased *ACAN* expression, which could both be ameliorated by ALK5 inhibition (Fig. 6a). Expression levels of hypertrophic *COL10A1* were strongly increased as a result of TGF-β supplementation (Fig. 6b), which was again inhibited by SB505124. Furthermore, pro-inflammatory IL-6 secretion was increased by supplementation of the calcification medium with TGF-β (Fig. 6c). Supplementation of TGF-β resulted in increased expression of *COL1A1* and *COL3A1*, which could be normalized by SB505124 (Fig. 6d). The increase in *COL1A1* and *COL3A1* was accompanied by a decrease in MMP-1, while MMP-13 expression was increased by TGF-β (Fig. 6e). These findings were confirmed by measuring MMP-1 and MMP-13 protein secretion in the culture medium (Fig. 6f). These data suggest that TGF-β-enhanced phosphate-driven calcification of human articular chondrocytes is associated by ECM remodeling.

## Discussion

In this study, we aimed to develop a phosphate-driven in vitro calcification model for human OA articular chondrocytes. This model allowed investigation of the yet unknown role of TGF-β in phosphate-driven calcification of OA-HACs. We observed a pro-calcifying role for TGF-β under phosphate-driven calcification conditions, which could be counteracted by the inhibition of ALK5 by SB505124.

Liberation of phosphate species from ATP and BGP relies on enzymatic hydrolysis. Generally, ATP is a substrate for ENPP1 which results in the formation of PPi while formation of Pi from BGP is generally the result of ALP-guided degradation. Apart from degradation of BGP, ALP may play a more central role in this model. Recent evidence has shown affinity of ALP for ATP, resulting in the generation of ADP and pro-calcifying Pi generation. Furthermore, ALP is capable of transforming PPi into extracellular Pi [12]. The use of ATP as sole phosphate donor in calcification assays dates back to the early 1990s, where 19-day-old chick embryo sterna rapidly degraded ATP, forming phosphate-containing minerals [29]. BGP as phosphate donor is common in osteogenic differentiation of mesenchymal stem cells [30] and vascular endothelial cell calcification [31]. BGP-aided mineralization has also been tested in chick embryo chondrocyte cultures for mineralization assays, where the hypertrophic chondrocyte phenotype was essential for the induction of calcification [32]. In contrast to previously published studies, our combinatorial approach utilizing multiple phosphate donors tends to yield a more nuanced perturbation of the physiological PPi/Pi balance in vitro. This nuance is created as a result of synthesis of PPi, originating from ATP addition, as well as Pi as a result of the BGP addition. Although the combinatorial use might initially create a more nuanced perturbation of the PPi/Pi ratio, we observed a greater calcification potential after 7 days. This enhanced calcification potential may therefore provide a better window to determine the influence of joint microenvironmental factors and potential anti-calcification drug candidates.

The addition of TGF-β under calcification conditions resulted in a more pronounced calcification of HAC ECM and formation of an alternative calcium-containing crystal species. Under control calcification conditions, ACP or α/β tricalcium phosphate crystals were formed. ACP has been demonstrated to be essential for early calcification [33], while tricalcium phosphate was described as a precursor for hydroxyapatite [34]. These results strongly hint toward a continuous process of calcification in the control calcification condition. Alternatively, the addition of TGF-β resulted in the formation of monetite or brushite. In contrast to monetite, brushite has been found in OA synovial fluid [35]. Furthermore, brushite is also a precursor for hydroxyapatite [36], a calcium-containing crystal whose presence has been detected in OA synovial fluid [37] and articular cartilage [2].

The difference in crystal species might be explained by the underlying cellular mechanisms under TGF-β-supplemented calcifying conditions. Intracellular Pi levels are among others regulated by type III sodium/inorganic phosphate transporters, such as SLC20A1 and SLC20A2. Dysregulation of these phosphate levels, thereby resulting in a high extracellular phosphate content, have been shown to activate the Raf/MEK/ER pathway in ATDC5 cells [38]. In our model, *SLC20A1* but not *SLC20A2* showed a TGF-β-induced upregulation. The TGF-β-mediated induction of *SLC20A1* may therefore result in an increased cellular uptake of Pi by chondrocytes thereby facilitating calcification. A TGF-β-mediated increase in Pi uptake was previously reported in ATDC5 cells [39]. Furthermore, this transporter has been hypothesized to play an important role in the uptake of Pi by matrix vesicles [40]; the presence of SLC20A1 on matrix vesicles remains to be elucidated [41, 42].

In contrast to the increased *SLC20A1* expression levels observed in the TGF-β-supplemented mineralization medium, no alteration in gene expression levels of *ANKH* was observed. These findings could indicate that the export of ATP, thereby increasing the extracellular production of PPi, is not enhanced under TGF-β-supplemented mineralizing conditions. Although the gene expression levels of this transporter were not differentially regulated after 7 days, shorter stimulations with TGF-β did induce increased expression of ANKH. Stimulation of rat chondrocytes for 24 h with TGF-β1 stimulated increased expression of ANKH [43]. This effect could be further enhanced by the addition of extracellular calcium. Moreover, in murine metatarsal bone cultures stimulation, TGF-β for 5 days resulted in increased expression of *ANKH* by chondrocytes residing in the growth plate [44].

The composition of the ECM plays a key role in the mineralization capacity. Indeed, we observed a steep TGF-β-induced increase in the expression of *COL10A1*. The role of hypertrophy in inducing cartilage mineralization, and more particularly the function of collagen type X, has been well described [45]. Due to the hexagonal molecular structure of collagen type X, it is hypothesized that it plays an essential role in the compartmentalization of the ECM [45]. The compartmentalized structure of the ECM is hypothesized to contain all the necessary elements which facilitate mineralization. This compartmentalization is suggested to allow the formation of a pericellular network consisting of all the necessary elements for the induction of mineralization in normal physiology as well as pathology. Due to the significant TGF-β-driven upregulation of *COL10A1*, more collagen type X is expected to be present in the matrix, thereby facilitating the TGF-β-aggravated mineral growth. Collagen type I aids mineralization by providing nucleation centers, the so-called hole zones [46]. These zones are surrounded by negatively charged amino acids capable of binding Ca^2+^. Blocking of these sites using a collagen type I-specific antibody was able to reduce mineral deposition in chick limb-bud mesenchymal cells [47]. The increased expression of *COL1A1* following TGF-β supplementation may therefore aid mineralization by increasing the number of nucleation centers. We also found an increased expression of *COL3A1*. Collagen type III is considered a key regulator of collagen fibrillary structure through co-assembling with collagen type I, thereby controlling the fibril diameter and collagen cross-links [48]. Overall, the increased expression of *COL10A1, COL1A1*, and *COL3A1* may therefore lead to an ECM that is more calcification-supportive.

A significant role in ECM remodeling is played by MMPs. We found decreased expression of *MMP-1* in response to TGF-β supplementation. The preferential substrate for MMP-1 is mainly collagen type I and III. Reduced MMP-1 expression may therefore result in more collagen type I deposition, thereby supporting a pro-mineralizing environment. In contrast to *MMP-1,* we found a significant increase of *MMP-13.* Increased activity of MMP-13 is expected to result in a reduction of Collagen type II deposition, thereby potentially diminishing the calcification inhibitory capacity that has previously been described for Collagen type II [49] and allowing mineralization of the matrix.

An indirect mechanism of the pro-calcifying function of TGF-β may be found in the increased secretion of pro-inflammatory factors. Literature reports a clear link between TGF-β stimulation and IL-6 secretion [24, 50]. The influence of pro-inflammatory factors on mineralization, more specifically inorganic phosphate homeostasis, has been shown for IL-6. Stimulation of human cartilage explants with IL-6 resulted in increased expression of *ANK*, *SLC20A1*, and *ENPP1* [51]. Indeed, we observed increased expression of IL-6 in response to TGF-β supplementation, which was associated with increased *SLC20A1* and *ENPP-1* expression.

In conclusion, we developed an in vitro phosphate-driven calcification model for human OA articular chondrocytes. Culturing in the presence of ATP and BGP potentiated mineral deposition. In this HAC calcification model, we found a potent pro-calcification role for TGF-β. TGF-β-dependent alterations in the chondrocyte hypertrophic and fibrotic phenotype, accompanied by decreased MMP-1 expression, may provide a rational for the TGF-β-driven chondrocyte calcification. Besides fundamental studies, our HAC calcification model can be used as a platform to determine the influence of joint environmental factors on phosphate-driven calcification and potentially aid the development of new OA-related calcification inhibitory molecular therapies.

## Supplementary Information

Below is the link to the electronic supplementary material.

Supplementary file1 (PDF 1180 KB)
