# Supplementary material for: TGF-β Enhances Phosphate-Driven Calcification of Human OA Articular Chondrocytes
Source: Calcif Tissue Int. 2025 Apr 2;116(1):57. doi: 10.1007/s00223-025-01365-x (PMC11965260; doi:10.1007/s00223-025-01365-x)
Supplement: Supplementary file 1 — Supplementary file1 (PDF 1180 KB) [file 223_2025_1365_MOESM1_ESM.pdf]

Supplementary Table 1
RT-qPCR primers

Gene	*Forward*	*Reverse*
SLC20A1	5'- AAGGCATTGCCCGAAATCT-3'	5'-TGCAAACGCTTAGGTAAGAAATGT-3'
SLC20A2	5'-CGGGCTGCATACGGAAGA-3'	5'-GGAGATGGGCGATTTCACA-3'
ANKH	5'-ACAGAGGCAGTGGCGATTTT-3'	5'-CCAGCCGTATGGCATGTG-3'
ENPP1	5'-GGCATTGCCCGAAATCTTT-3'	5'-TGCAAACGCTTAGGTAAGAAATGT-3'
ALPL	5’-CCGTGGCAACTCTATCTTTGG-3’	5’-CAGGCCCATTGCCATACAG-3’
COL2A1	5’-TGGGTGTTCTATTTATTTATTGTCTTCCT-3’	5’-GCGTTGGACTCACACCAGTTAGT-3’
ACAN	5’-GCAGCTGGGCGTTGTCA-3’	5’-TGAGTACAGGAGGCTTGAGGACT-3’
COL10A1	5'-ATGATGAATACACCAAAGGCTACCT-3'	5'-ACGCACACCTGGTCATTTTCTG-3'
IL-6	5’-TGTAGCCGCCCCACACA-3’	5’-GGATGTACCGAATTTGTTTGTCAA-3’
COL1A1	5’-TGGAGAGTACTGGATTGACCC-C-3’	5’-TGCAGAAGACTTTGATGGCATC-3’
COL3A1	5'-CCGTTCTCTGCGATGACATAATAT-3'	5'-GCACAACATTCTCCAAATGGAA-3'
MMP-1	5'-TGAGCATCCCCTCCAATACC-3’	5'-GATGGACCTGGAGGAAATCTTG-3’
MMP-13	5'-CTTCACGATGGCATTGCTGAC-3'	5'-CGCCATGCTCCTTAATTCCA-3'
PPIA	5'-TTCCTCCTTTCACAGAATTATTCCA-3’	5'- CCGCCAGTGCCATTATGG-3’

Supplementary figure 1

LDH activity durnig chondrocyte mineralization`

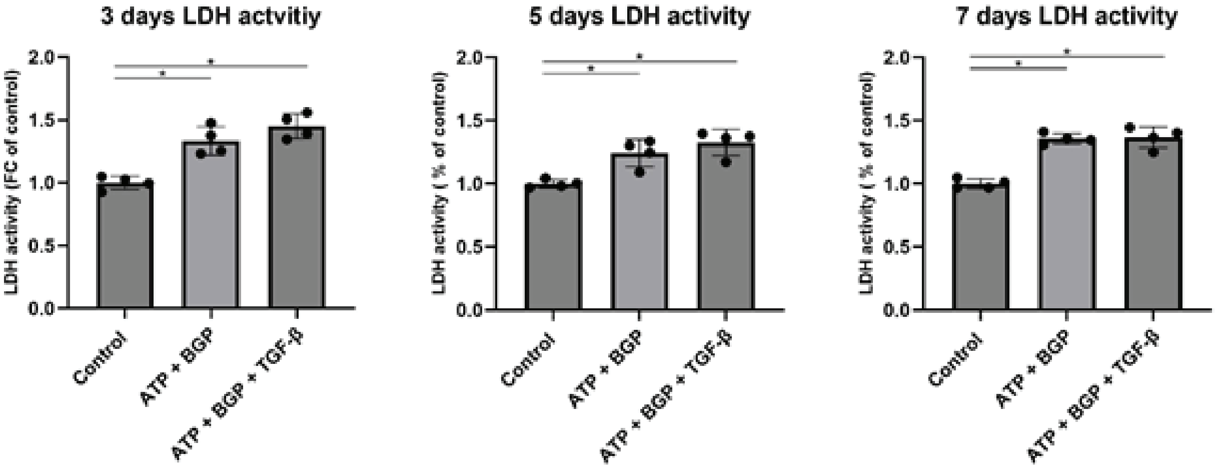

LDH activity was determined after 3, 5 and 7 days of exposure to either calcification medium alone or calcification medium supplemented with 10 ng/ml TGF-β (n=4 biological replicates). Determination of statistical significance was performed with use of an unpaired Students T-Test. Data are presented as mean ± SD. * P.value < 0.05.

Supplementary figure 2

Characterization of spontaneous mineralization in absence of cells

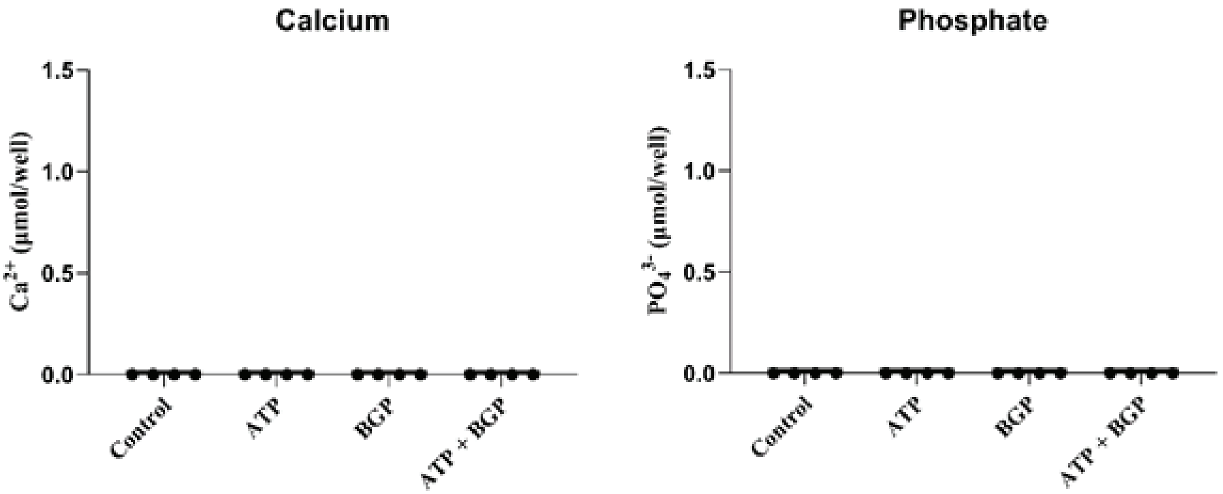

Formation of calcified nodules was investigated in a cell-free manner. Cell-free wells containing culture medium supplemented with either ATP, BGP or ATP plus BGP were incubated for 7 days with refreshment at day 3, 5 and 7. After 7 days, the wells were treated with 0.1 M of HCl to lyse any crystals formed. In this lysate, calcium and phosphate presence was determined with use of colorimetric assays. For all combinations, no crystal deposition was detected. Data are presented as Mean ± SD.

Supplementary figure 3

SEM-EDX analysis of control calcification condition

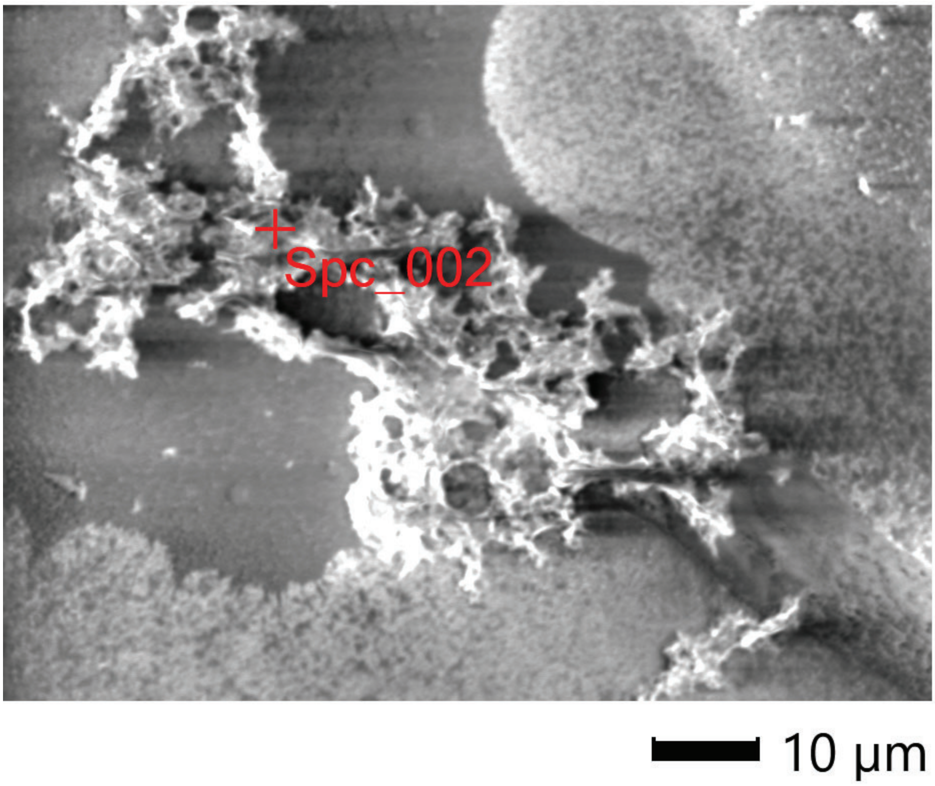

Signal BED-C
Landing Voltage 10.0 kV
WD 11.2 mm
Magnification x1,500
Vacuum Mode LowVacuum

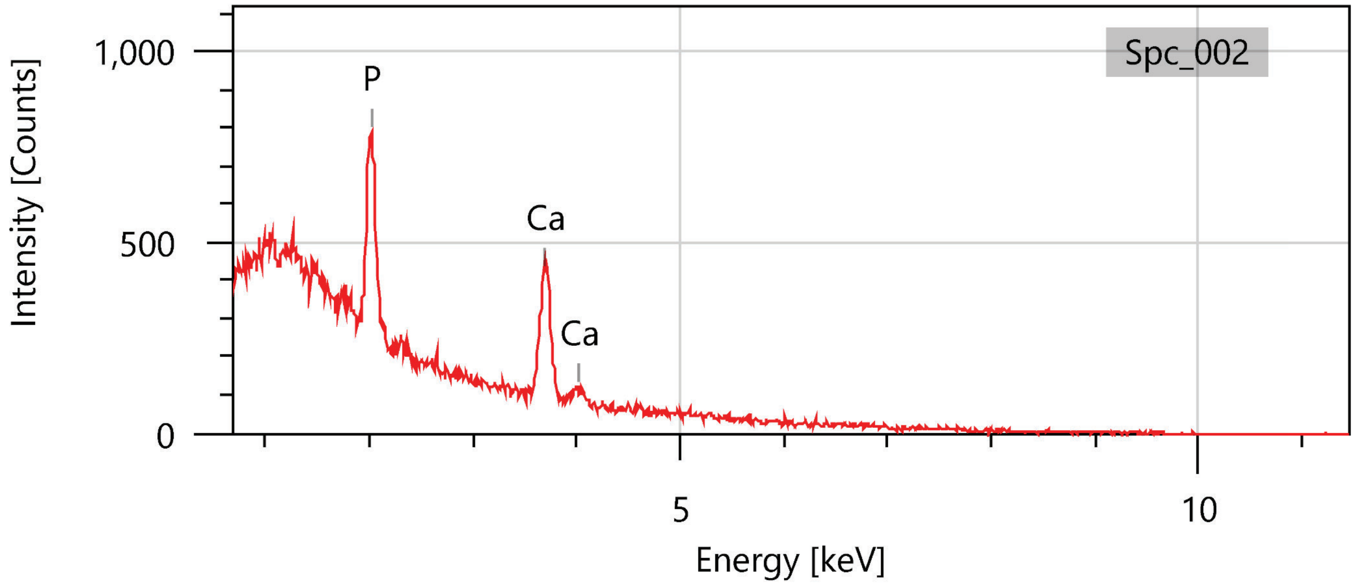

Element	Line	Mass%	Atom%
C	K	89.18±0.07	92.75±0.07
O	K	8.05±0.05	6.29±0.04
P	K	1.15±0.02	0.46±0.01
Ca	K	1.63±0.04	0.51±0.01
Total		100.00	100.00
Spc_002			Fitting ratio 0.0778

Supplementary figure 4

SEM-EDX analysis of TGF-β supplemented calcification condition

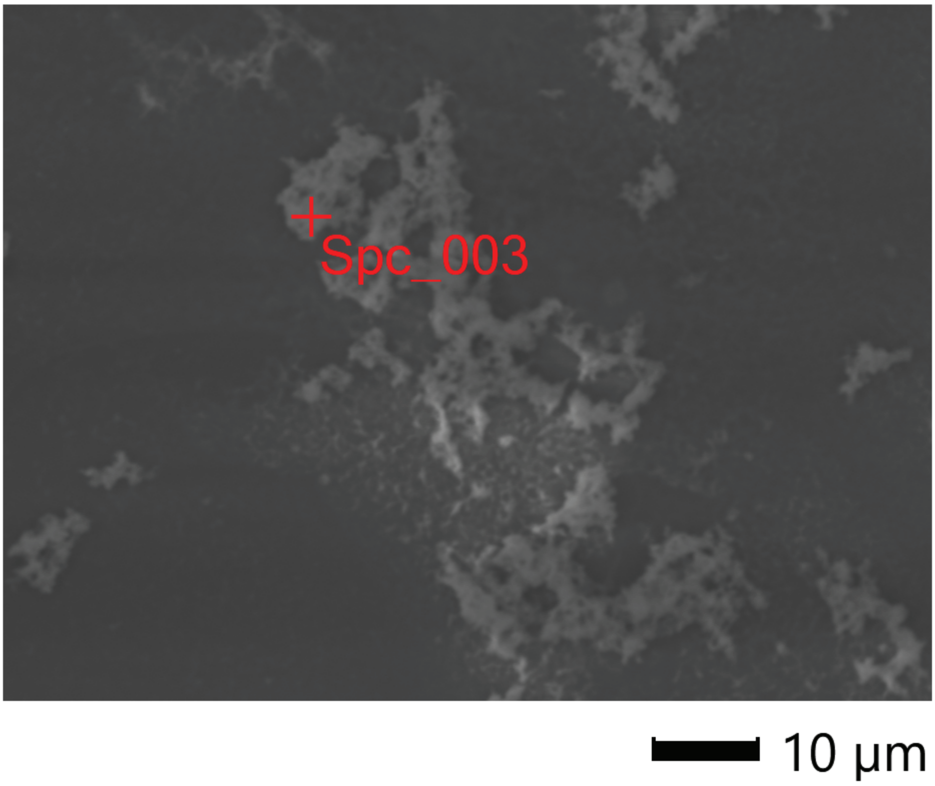

Signal BED-C
Landing Voltage 10.0 kV
WD 10.0 mm
Magnification x1,500
Vacuum Mode LowVacuum

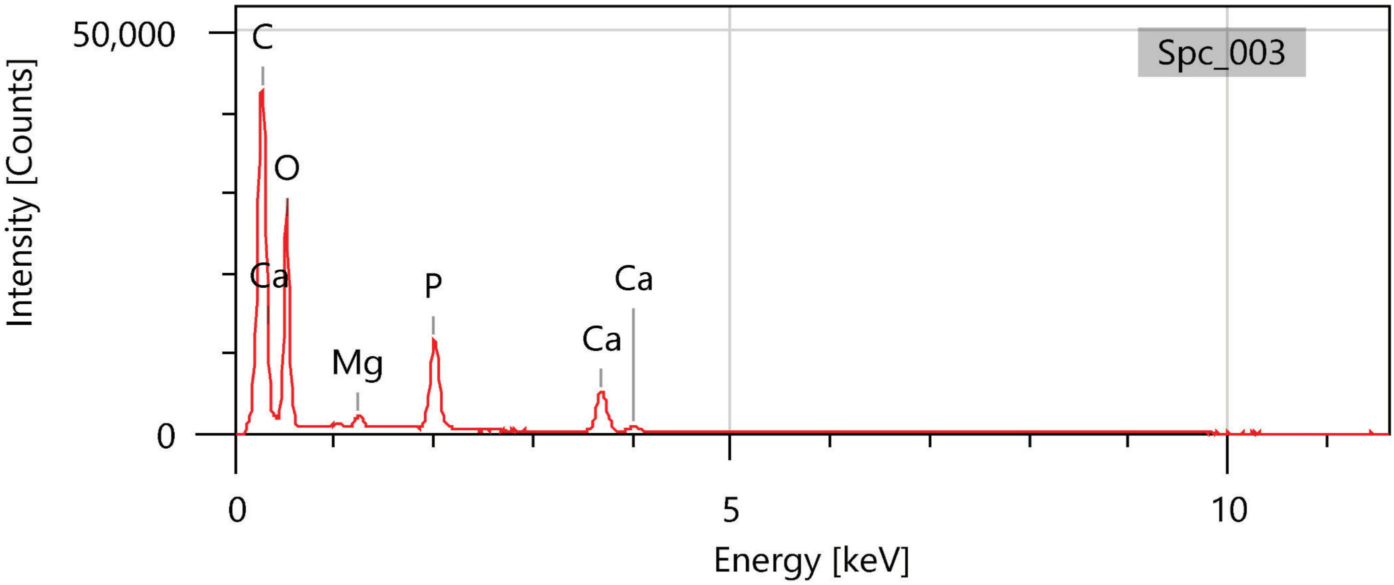

Element	Line	Mass%	Atom%
C	K	50.58±0.05	64.62±0.06
O	K	26.11±0.06	25.04±0.06
Mg	K	0.71±0.01	0.45±0.01
P	K	11.07±0.05	5.48±0.02
Ca	K	11.52±0.07	4.41±0.03
Total		100.00	100.00
Spc_003			Fitting ratio 0.0344
